# INFECTION CONTROL ADVOCATE AND RESIDENT EDUCATION (ICARE): PERSON-CENTERED INFECTION PREVENTION FOR QUALITY CARE

**DOI:** 10.1093/geroni/igad104.1579

**Published:** 2023-12-21

**Authors:** Diana Cervantes

**Affiliations:** University of North Texas Health Science Center, Fort Worth, Texas, United States

## Abstract

Applying infection prevention and control (IPC) measures while ensuring person-centered care (PCC) remains a major challenge in nursing homes. PCC focuses on resident rights to engage and empower nursing community residents, families, advocates, and staff to deliver quality care. To address this gap, The Infection Control Advocate and Resident Education (ICARE) program was developed for nursing home residents and families. ICARE is a step-based program which begins with educating participants on IPC and resident rights through seven web-based modules. The program encourages support and collaboration of nursing home advocate organizations including the long-term care ombudsman program, quality care organizations, and public health departments to assist residents and families in becoming more engaged and empowered in quality care beginning with infection prevention and control. Utilizing ICARE program materials, participants progress to engagement by learning how quality care is provided in their nursing home community, with the goal of empowering residents to actively participate in quality care improvement through quality improvement projects. The ICARE Program assists nursing home advocate organizations to identify opportunities to incorporate elements of infection control, quality care, and resident rights in current procedures and activities. The overall program design and theory of the ICARE program will be presented to provide a foundation for the creation of similar programs.

